# Supplementary material for: BIG Modulates Stem Cell Niche and Meristem Development via SCR/SHR Pathway in Arabidopsis Roots
Source: Int J Mol Sci. 2022 Jun 17;23(12):6784. doi: 10.3390/ijms23126784 (PMC9224481; doi:10.3390/ijms23126784)
Supplement: Supplementary file 1 [file ijms-23-06784-s001.zip › ijms-1783470-supplementary.pdf]

BIG modulates stem cell niche and meristem development via SCR/SHR pathway in Arabidopsis roots

Zhongming Liu [1], Ruo-Xi Zhang [1], Wen Duan [1], Baoping Xue [1], Xinyue Pan [1], Shuangchen Li [1], Peng Sun [1], Limin Pi [3*] and Yun-Kuan Liang [1,2*]

Supplementary Materials

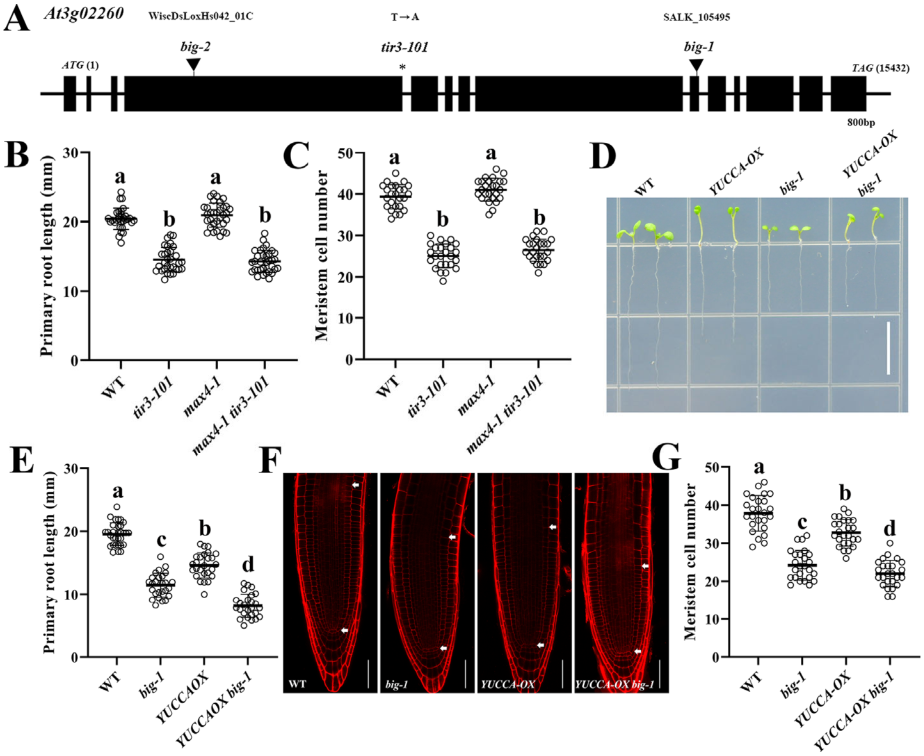

Figure S1: Increasing auxin does not restore the root phenotype of *big-1*. (A) Schematic diagram of BIG. The triangle represents the T-DNA insertion position, and the asterisk represents the point mutation position. (B) Primary root length of WT, *tir3-101*, *max4-1*, and *max4-1 tir3-101* at 5 DAG, Values are means ± SD (n ≥ 30). One-way ANOVA, Tukey's multiple comparisons test, P < 0.01. (C) Quantification of meristem cell number of WT, *tir3-101*, *max4-1* and *max4-1 tir3-101* seedlings at 5 DAG. Values are means ± SD (n ≥ 25). One-way ANOVA, Tukey's multiple comparisons test, P < 0.01. (D) Images of the indicated genotypes WT, *big-1*, *YUCCA-OX*, and *YUCCA-OX big-1* at 5 DAG. Scale bar, 5 mm. (E) Primary root length of WT, *big-1*, *YUCCA-OX*, and *YUCCA-OX big-1* at 5 DAG, Values are means ± SD (n ≥ 30). One-way ANOVA, Tukey's multiple comparisons test, P < 0.01. (F) Root apical phenotypes of WT, *big-1*, *YUCCA-OX*, and *YUCCA-OX big-1* seedlings at 5 DAG. Scale bars, 50 µm. (G) Quantification of meristem cell number of WT, *big-1*, *YUCCA-OX*, and *YUCCA-OX big-1* seedlings at 5 DAG. Values are means ± SD (n ≥ 25). One-way ANOVA, Tukey's multiple comparisons test, P < 0.01.

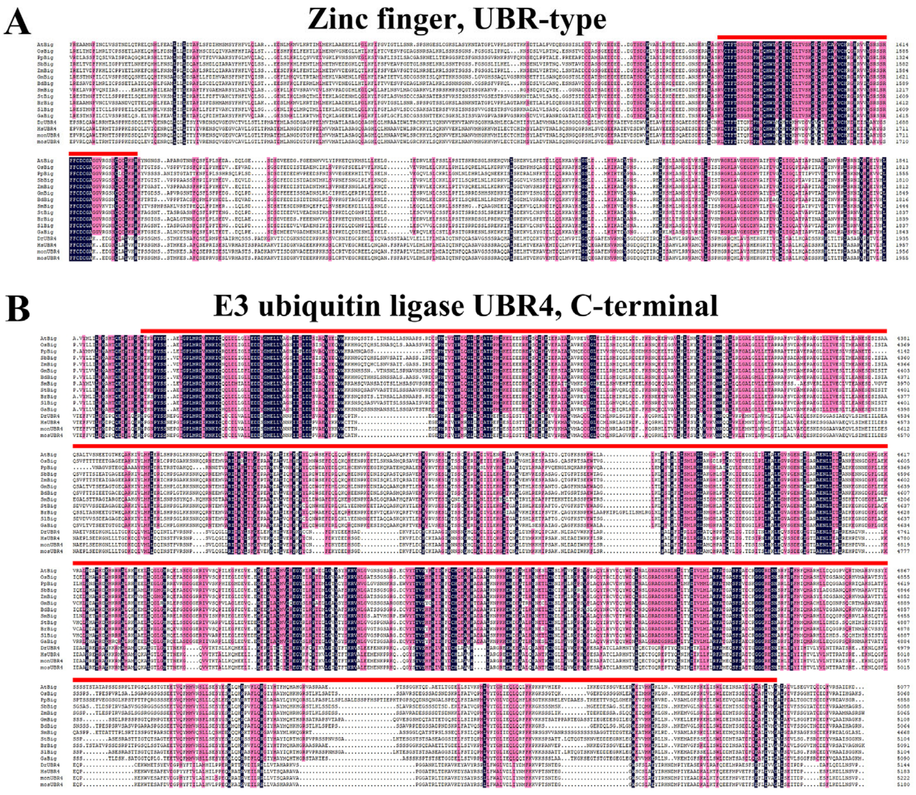

Figure S2: The highly conserved Arabidopsis BIG. (A) Alignment of BIG proteins among different species. Identical amino acids are indicated in the same color. The red line represents the conserved UBR domain and E3 ubiquitin ligase domain.

Table S1. Primers used in this study.

Name	Sequences (5'-3')
WOX5-q-FP	GATTGTCAAGAGGAAGAGAAGGTGA
WOX5-q-RP	AGCTTAATCGAAGATCTAATGGCG
SHR-q-FP	TCCAAGGACGAGCAACGAGAGGT
SHR-q-RP	CCGGCGGCATCAGGACACT
SCR-q-FP	TTCCCGCCGTCCAAACAAATA
SCR-q-RP	TGCGCTGAGGTCCCGTAAGG
PLT1-q-FP	ACATCGATGGACTGGTCGATATGAG
PLT1-q-RP	TGAAGGACCCCAGTACTTAAGTGCA
PLT2-q-FP	TACCTTATAACGACATTCATCAA
PLT2-q-RP	GAGCATTAGAGGCACAAG
PIN1-q-FP	TTACTGTTCGTCGTTCTA
PIN1-q-RP	TATATCTCAGCGTTGGTTA
PIN2-q-FP	GAAGAATGCTATGACCAGAG
PIN2-q-RP	TTGTCGTGAGGAGGAATA
PIN3-q-FP	TTCGGAGGAGAACAACAA
PIN3-q-RP	TGGAATTTGGAGCAAGTTT

PIN7-q-FP	GAGAATCATCATCATGTAGG
PIN7-q-RP	GGCTCCATTATCAACTTG
Actin7-q-FP	CCATTCAGGCCGTTCTTTC
Actin7-q-RP	CGTTCTGCGGTAGTGGTGA
